# Targeting Myeloid-Derived Suppressor Cells to Bypass Tumor-Induced Immunosuppression

**DOI:** 10.3389/fimmu.2018.00398

**Published:** 2018-03-02

**Authors:** Viktor Fleming, Xiaoying Hu, Rebekka Weber, Vasyl Nagibin, Christopher Groth, Peter Altevogt, Jochen Utikal, Viktor Umansky

**Affiliations:** ^1^Skin Cancer Unit, German Cancer Research Center (DKFZ), Heidelberg, Germany; ^2^Department of Dermatology, Venereology and Allergology, University Medical Center Mannheim, Heidelberg University, Mannheim, Germany

**Keywords:** myeloid-derived suppressor cells, immunosuppression, cancer immunotherapy, tumor microenvironment, therapeutic targeting

## Abstract

The immune system has many sophisticated mechanisms to balance an extensive immune response. Distinct immunosuppressive cells could protect from excessive tissue damage and autoimmune disorders. Tumor cells take an advantage of those immunosuppressive mechanisms and establish a strongly immunosuppressive tumor microenvironment (TME), which inhibits antitumor immune responses, supporting the disease progression. Myeloid-derived suppressor cells (MDSC) play a crucial role in this immunosuppressive TME. Those cells represent a heterogeneous population of immature myeloid cells with a strong immunosuppressive potential. They inhibit an antitumor reactivity of T cells and NK cells. Furthermore, they promote angiogenesis, establish pre-metastatic niches, and recruit other immunosuppressive cells such as regulatory T cells. Accumulating evidences demonstrated that the enrichment and activation of MDSC correlated with tumor progression, recurrence, and negative clinical outcome. In the last few years, various preclinical studies and clinical trials targeting MDSC showed promising results. In this review, we discuss different therapeutic approaches on MDSC targeting to overcome immunosuppressive TME and enhance the efficiency of current tumor immunotherapies.

## Introduction

Immunosuppression is a hallmark of most cancer entities and is pivotal for cancer growth and progression (1, 2). In recent years, accumulating data highlighted myeloid-derived suppressor cells (MDSC) as one of the main driver of an immunosuppressive tumor microenvironment (TME) (3). Their accumulation and activation correlated with tumor progression, metastasis, and recurrence of many types of tumors. In addition, the efficacy of immunotherapy was negatively correlated with an increased MDSC frequency and activity (4, 5). Therefore, targeting MDSC becomes a promising treatment approach to overcome tumor progression and tumor-mediated immunosuppression.

**Figure 1:**
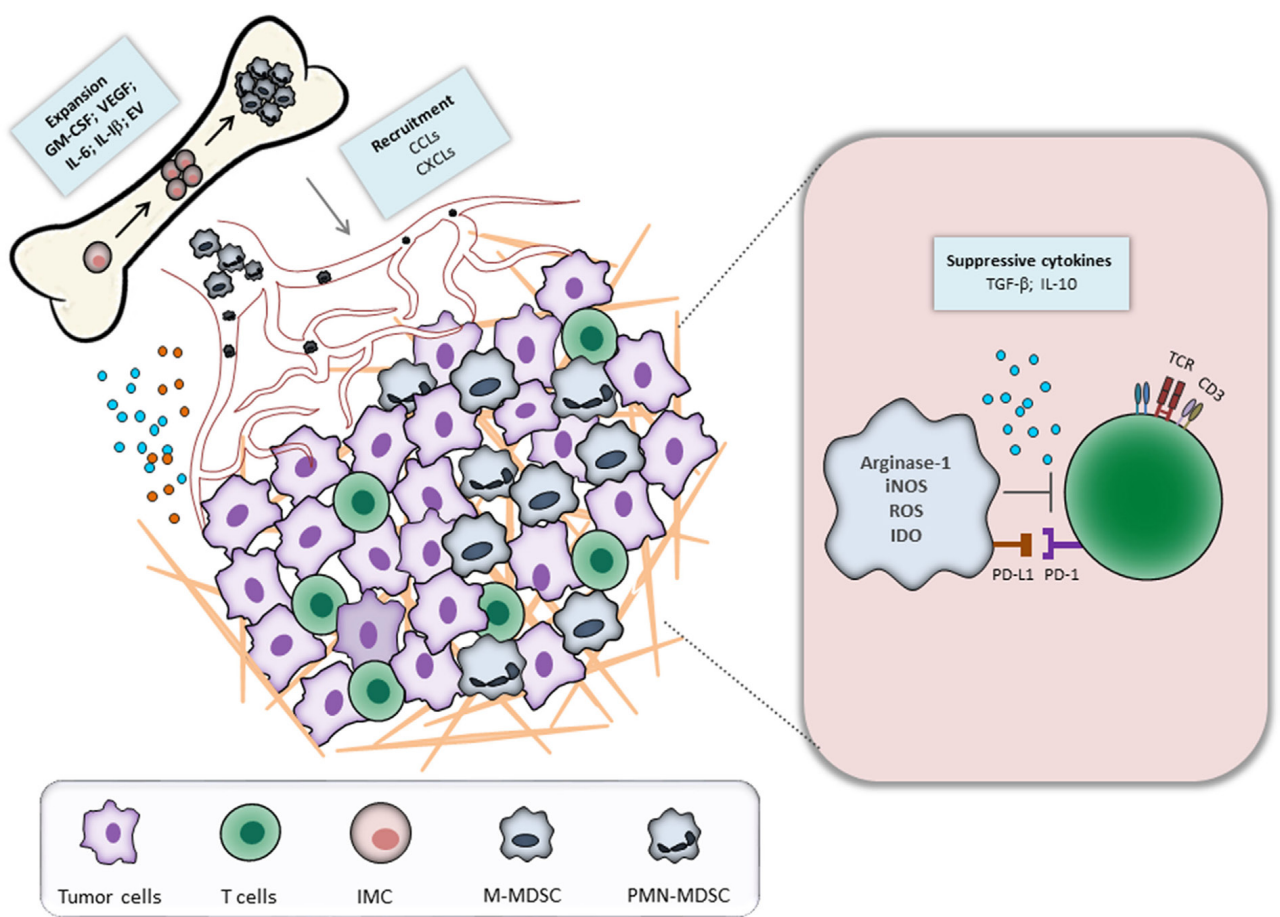
Myeloid-derived suppressor cells (MDSC) recruitment and activation during tumor progression. Tumor and immune cells constantly release inflammatory mediators, leading to the dysregulation of normal myelopoiesis and to the conversion of immature myeloid cells (IMC) into MDSC in the bone marrow. The latter cells expand and migrate to the tumor site through the interaction between CCR and respective chemokines (CCL). In the tumor microenvironment, MDSC are activated and strongly inhibit an antitumor reactivity of T cells *via* various mechanisms.

Myeloid-derived suppressor cells represent a heterogeneous population of immature myeloid cells (IMC) that fail to terminally differentiate and exhibit a strong capacity to suppress the functions of T and NK cells (6–9). Under healthy conditions, IMC differentiate into macrophages, dendritic cells (DCs), or granulocytes. During an acute inflammation, IMC expand and differentiate mainly into monocytes and activated neutrophils (7). This process, known as myelopoiesis, is essential to protect the host from pathological conditions. In contrast to acute inflammation, chronic inflammation and cancer are characterized by a persistent release of signals of low stimulatory intensity (10–12). Although these stimuli still activate myelopoiesis, the accumulating IMC fail to completely differentiate into activated neutrophils and monocytes. Instead, the long-term inflammatory signals create conditions for the expansion and activation of MDSC (13, 14). They migrate to the site of inflammation, lymphoid organs, and pre-metastatic niches and promote tumor progression by immunological and non-immunological mechanisms (15). Figure 1 illustrates the biology and functions of MDSC during tumor progression.

## Phenotype of MDSC

Myeloid-derived suppressor cells consist of two major subpopulations, which are traditionally described by their phenotypical and morphological characteristics. The first population is called monocytic MDSC (M-MDSC), whereas the second is polymorphonuclear MDSC (PMN-MDSC) (8), which was previously known as granulocytic MDSC (6). Both MDSC subsets can be found under pathological conditions in the bone marrow, spleen, lung, peripheral blood, and tumor tissue; in most cancer entities, PMN-MDSC represent more than 80% of all MDSC (16). In mice, M-MDSC are defined as CD11b^+^Ly6G^−^Ly6C^high^ and share phenotypical and morphological characteristics with monocytes. PMN-MDSC are described as CD11b^+^Ly6G^high^Ly6C^low^ cells and resemble neutrophils (16, 17). In human, M-MDSC are defined as CD11b^+^CD14^+^CD15^−^HLA-DR^low/−^ cells. Due to the low or absence of the HLA-DR expression, M-MDSC they can be distinguished from monocytes. Human PMN-MDSC are characterized as CD11b^+^CD14^−^CD15^+^HLA-DR^−^ or CD11b^+^CD14^−^CD66b^+^ (17, 18). In addition, a subset of more immature human MDSC characterized as Lin^−^ (including CD3, CD14, CD15, CD19, CD56) HLA-DR^−^CD33^+^ cells was defined as early-stage MDSC (eMDSC) (17). At the moment, the mouse equivalent of eMDSC is not clearly determined. Recently, a new marker for human PMN-MDSC has been proposed; they were found to express lectin-type oxidized LDL receptor-1 (LOX-1) that can discriminate them from neutrophils (19).

## Conversion of IMC into MDSC by Tumor-Derived Extracellular Vesicles (EV)

Expansion and activation of MDSC could be stimulated by many soluble factors, which are predominately released within the TME by tumor and immune cells (20). Specifically, granulocyte-macrophage colony-stimulating factor (GM-CSF), granulocyte CSF, macrophage CSF, stem cell factor, transforming growth factor (TGF)-β, tumor necrosis factor (TNF)-α, vascular endothelial growth factor (VEGF), prostaglandin E2, cyclooxygenase 2, S100A9, S100A8, interleukin (IL)-1β, IL-6, and IL-10 are considered to be crucial for MDSC expansion (6, 8, 21–23). Furthermore, tumor cells can stimulate the secretion of these inflammatory mediators by cancer-associated fibroblasts and vice versa leading to an autocrine loop, which promotes tumor growth by converting myeloid cells into MDSC (20).

In addition to soluble inflammatory factors, tumor-derived EV could contribute to the generation of MDSC. EV consist of microvesicles that are created by the outward budding of the plasma membrane and exosomes, which are generated through the endosomal system (24). Due to their phospholipid bilayer, EV are stable vehicles to carry biological active molecules (25). It was shown that tumor-derived EV are predominately taken up by MDSC (26). After the uptake of EV derived from a Lewis lung carcinoma (LLC) and glioma, MDSC displayed an increased expression of immunosuppressive molecules like arginase-1 (ARG1) and programmed death ligand 1 (PD-L1) (26). Filipazzi et al. (27) demonstrated that CD14^+^ monocytes lost the expression of HLA-DR and acquired an immunosuppressive activity upon EV uptake. In contract, EV from healthy donors were not able to convert monocytes into MDSC-like cells (27). Several studies showed that EV trigger toll-like receptor (TLR) signaling in myeloid cells. THP-1 monocytic cell line showed increased production of inflammatory molecules like IL-1β, IL-6, and TNF-α upon the EV treatment, which was due to TLR2 and TLR4 signaling (28, 29). Chalmin et al. (30) demonstrated that tumor-derived EV triggered the expansion and activation of murine and human MDSC *via* HSP72 that stimulated TLR2 signaling. Furthermore, by using the B16 transplantable melanoma model, it was shown that tumor EV could facilitate formation of metastasis through the transfer of the Met receptor tyrosine kinase to bone marrow cells (31). As the bone marrow cells were not further characterized, it is conceivable that such melanoma-derived EV converted bone marrow-derived IMC into potent MDSC.

## Immunosuppression Induced by MDSC

Myeloid-derived suppressor cells use a broad range of suppressive molecules to inhibit antitumor reactivity of immune cells, supporting thereby tumor growth and metastasis. By inhibiting the activity of tumor-infiltrating lymphocytes, MDSC show their extraordinary potential of silencing the immune response (6–11, 16–18, 32, 33). One of the main immunosuppressive mediators is ARG1, which is an essential enzyme for the urea cycle (34, 35). It converts l-arginine into l-ornithine and urea, leading to the depletion of l-arginine. The lack of l-arginine causes a translational blockade in infiltrating T cells leading to cell cycle arrest in G_0_-G_1_ (36). Furthermore, T cells become anergic due to the downregulation of the T cell receptor (TCR) ζ-chain, which is essential for TCR signaling (37). Besides ARG1, MDSC express also of inducible nitric oxide synthase (iNOS), which also catabolize l-arginine. The main product of the reaction is nitric oxide (NO) that could induce T cell anergy (16) and nitrosylate important mediators of the IL-2 pathway (38). MDSC express also elevated levels of indoleamine 2,3-dioxygenase (IDO) that degrade l-tryptophan into *N*-formylkynurenine. The lack of tryptophan results in the cell cycle arrest in T cells and induces anergy (39). Moreover, tryptophan starvation is known to drive the differentiation of CD4^+^ T cells into immunosuppressive regulatory T cells (Treg) (40). Kynurenine and 3-hydroxykynurenine, the products of IDO activity, exert also immunosuppressive functions, inhibiting effector T cell survival and proliferation (41). In addition, kynurenine drives the differentiation of CD4^+^ T cells into Treg and induces apoptosis in thymocytes (42, 43). Kynurenine was also reported to dampen NK cell function and proliferation (44). Furthermore, reactive oxygen species (ROS) produced by MDSC in high concentrations were shown to induce T cell apoptosis (9, 11, 16) In addition, ROS was demonstrated to downregulate the expression of TCR ζ-chain, leading to impaired TCR signaling (10, 16, 17). Reacting with NO, ROS form peroxynitrite, which nitrosylates the TCR, resulting in T cell anergy (45). MDSC also secrete immunosuppressive cytokines and growth factors such as TGF-β and IL-10 that reduce antitumor activity of effector T cells and recruit Treg (46, 47).

It has been recently described that MDSC could exert their immunosuppressive effects *via* upregulation of PD-L1 (48, 49). Upon the binding of PD-L1 to the PD-1 receptor expressed on T cells, they become anergic, losing their ability to produce interferon (IFN)-γ and IL-2 (48). Moreover, MDSC were shown to express the death receptor CD95 and induce T cell apoptosis *via* CD95 ligand expressed on activated T cells (50).

## Non-Immunological Ways of Promoting Tumor Progression

In addition to the establishment of an immunosuppressive TME, MDSC could promote tumor progression by non-immunological mechanisms (51). In particular, MDSC produce large amounts of matrix metalloproteinases (MMP), especially MMP9, which process the extracellular matrix and basal membrane and enable the tumor to leave the tissue, to enter the blood stream, and migrate to the site of later metastasis (52). It was shown that the pre-metastatic niche is performed before the tumor cells enter the blood stream (53). This process is still not fully understood but studies have confirmed that MDSC play an essential role (9, 54). It was found that MDSC accumulated in pre-metastatic niches with the help of monocyte chemoattractant protein-1 that dampens the activity of NK cells, which are also preferably found in the pre-metastatic niche (55). In addition, it was reported that MDSC produce MMP9 within the pre-metastatic niche, facilitating the penetration of metastatic cells (56). A further hallmark of tumor progression is angiogenesis that is crucial for the nutrition, vasculature, and dissemination of the tumor (57). MDSC promote angiogenesis by secreting elevated levels of VEGF and basic fibroblast growth factor (bFGF) (58). It was reported that blocking of angiogenesis resulted in the inhibition of tumor migration and formation of metastasis (59).

## Correlation between Tumor Burden, Resistance to Immunotherapy, and MDSC

The expansion of MDSC has been demonstrated in many types of human tumors (6, 7). Moreover, elevated levels of MDSC were found not only in solid tumors but also in blood of non-Hodgkin lymphoma and multiple myeloma patients (18). Importantly, the frequency of circulating MDSC was found to correlate with the disease stage. It was reported that patients with stage III and IV hepatocellular carcinoma, melanoma, non-small cell lung cancer, pancreatic, esophageal, gastric, and bladder cancer had higher frequencies of MDSC in the peripheral blood as compared to stage I and II patients (60–63). In addition, an association between MDSC numbers and clinical response to radio-, chemo-, and immunotherapy was reported (64). Several recent studies described that in melanoma patients treated with the immune checkpoint inhibitor, ipilimumab, decreased amounts and immunosuppressive functionality of both M- and PMN-MDSC correlated with beneficial therapeutic effects (65–68). Altogether, these studies show that MDSC could be not only promising biomarkers for the survival of patients and the treatment efficacy but also could serve as a valuable target in combined immunotherapy of cancer patients.

## MDSC Targeting in Cancer

**Figure 2:**
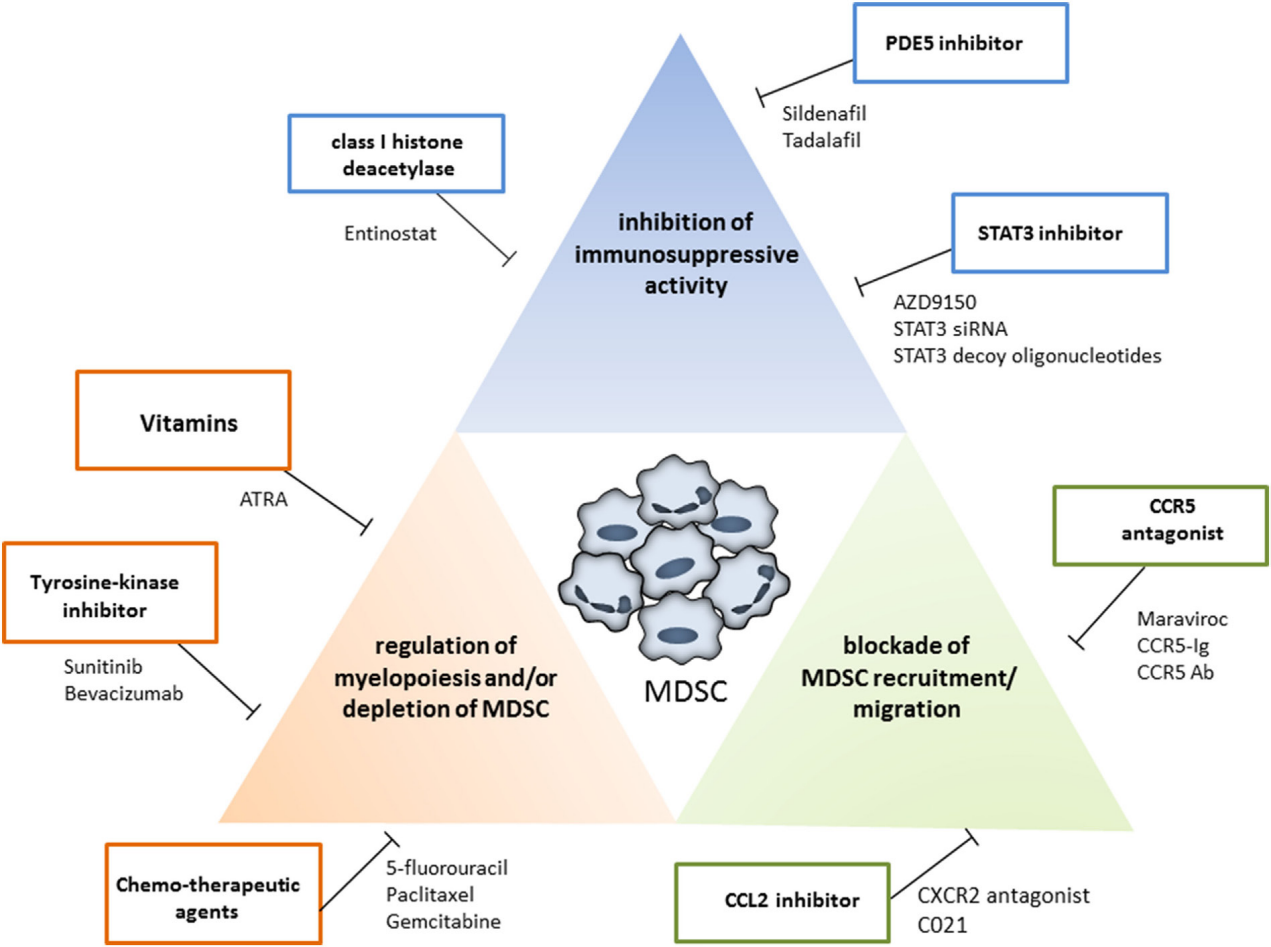
Strategies for myeloid-derived suppressor cells (MDSC) targeting. The MDSC modulation could be achieved by the inhibition of their immunosuppressive activity (blue box), by the blockade of MDSC recruitment to the tumor site (green box), and by the regulation of myelopoiesis and/or depletion of MDSC (red box). Examples for each therapeutic approach are shown.

In recent years, increasing numbers of preclinical and clinical studies were performed to target MDSC with beneficial effects, resulting in the tumor growth inhibition and the survival prolongation. The MDSC modulation was achieved by (i) the inhibition of their immunosuppressive activity; (ii) the blockade of MDSC recruitment to the tumor site; and (iii) the regulation of myelopoiesis and/or depletion of MDSC in the tumor-bearing hosts (Figure 2). Ongoing clinical trials are summarized in Table 1.

**Table 1:** Ongoing clinical trials to target myeloid-derived suppressor cells (MDSC) in cancer patients.

No.	Title	Disease or conditions	Interventions	Trial number
1	MDSC and chronic myeloid leukemia	Chronic myeloid leukemia	Imatinib	NCT03214718

2	Depletion of MDSC to enhance anti-PD-1 therapy	Non-small cell lung cancer (NSCLC), stage IIIB	Nivolumab	NCT03302247
			Nivolumab + Gemcitabine	

3	MDSC and checkpoint immune regulators’ expression in allogeneic SCT Using Flu-Bu-ATG	Leukemia, myelodysplastic syndromes	Fludarabine, Busulfan	NCT02916979
			Methotrexate	

4	MDSC control by signal regulatory protein-alpha: investigation in hepatocellular carcinoma	Hepatocellular carcinoma	Therapy-independent collection of human samples	NCT02868255

5	Myeloid-derived suppressor cells clinical assay in finding kidney cancer	Metastatic and recurrent renal cell cancer	Computed tomography, cytology specimen collection, laboratory biomarker analysis, magnetic resonance imaging	NCT02664883

6	Capecitabine + bevacizumab in patients with recurrent glioblastoma	Glioblastoma	Capecitabine	NCT02669173
			Bevacizumab	

7	Dendritic cell (DC) vaccine with or without gemcitabine. pre-treatment for adults and children with sarcoma	Sarcoma	Gemcitabine	NCT01803152
		Soft tissue sarcoma		
		Bone sarcoma	DCs vaccine	

8	SX-682 treatment in subjects with metastatic melanoma concurrently treated with pembrolizumab	Melanoma stage III	SX-682	NCT03161431
		Melanoma stage IV	Pembrolizumab	

9	PDE5 inhibition *via* tadalafil to enhance antitumor Mucin 1 vaccine efficacy in patients with HNSCC	Head and neck squamous cell carcinoma	Tadalafil	NCT02544880
			Anti-MUC1 vaccine	
			Anti-influenza vaccine	

10	Phase II trial of EP4 receptor antagonist, AAT-007 (RQ-07; CJ-023,423) in advanced solid tumors	Prostate cancer	RQ-00000007	NCT02538432
		NSCLC		
		Breast cancer	Gemcitabine	

11	MDSC clinical assay in finding and monitoring cancer cells in blood and urine samples from patients with or without localized or metastatic bladder cancer	Stage II bladder cancer	Cytology specimen collection procedure, laboratory biomarker analysis	NCT02735512
		Stage III bladder cancer		

12	RTA 408 capsules in patients with melanoma—REVEAL	Melanoma	Omaveloxolone	NCT02259231
		Unresectable (stage III) melanoma	Ipilimumab	
		Metastatic (stage IV)	Nivolumab	

13	PDL-1 expression on circulating tumor cells in NSCLC	Lung cancer	Blood sample collection for CTC and MDSC analysis	NCT02827344

14	Effect of Astragalus-based formula: Qingshu-Yiqi-Tang on modulating immune alterations in lung cancer patients	Non-small-cell lung carcinoma	Astagalus-based formula: Qingshu-Yiqi-Tang	NCT01802021

16	A phase II trial of tadalafil in patients with squamous cell carcinoma of the upper aero-digestive tract	Head and neck squamous cell carcinoma	Tadalafil	NCT01697800

17	Relevance of peripheral cells in the pathophysiology of chronic myelomonocytic leukemia	Chronic myelomonocytic leukemia	Clinical data collection	NCT03280888

18	Histamine receptor 2 antagonists as enhancers of antitumor immunity	Cancer	Ranitidine	NCT03145012

19	Preoperative nutrition with immune enhancing nutritional supplement (immunomodulation)	Pancreatic adenocarcinoma	Dietary supplement: Nestle IMPACT advanced recovery and Nestle Boost high protein drink	NCT02838966

20	A study of RGX-104 in patients with advanced solid malignancies and lymphoma	Malignant neoplasms	RGX-104	NCT02922764

21	Determination of immune phenotype in glioblastoma patients	Glioblastoma multiforme	Surgery	NCT02751138

22	Academia Sinica Investigator Award 2010	Breast cancer	Unknown	NCT01287468

23	The “Fuzzing” therapy of TCM to improve the survival quality of early-stage NSCLC by intervening the CTCs	NSCLC	JinFuKang	NCT02603003
			Cisplatin	
			Pemetrexed	

24	Antibody DS-8273a administered in combination with nivolumab in subjects with advanced colorectal cancer	Colorectal neoplasm	DS-8273a + nivolumab	NCT02991196

25	Study to assess safety and immune response of stage IIB-IV resected melanoma after treatment with MAGE-A3 ASCI	Melanoma	recMAGE-A3 + AS15 ASCI	NCT01425749

26	Potentiation of cetuximab by regulatory T cells depletion with CSA in advanced head and neck cancer	Head and neck squamous cell carcinoma	Cyclophosphamide	NCT01581970
			Cetuximab	

27	IMA970A plus CV8102 in very early, early and intermediate stage hepatocellular carcinoma patients	Hepatocellular carcinoma	IMA970A, CV8102, Cyclophosphamide	NCT03203005

28	Intensive locoregional chemoimmunotherapy for recurrent ovarian cancer plus intranodal DC vaccines	Cancer of ovary	Cisplatin + celecoxib + DC vaccine, cisplatin + CKM + celecoxib + DC vaccine	NCT02432378

29	Trial of SBRT with concurrent ipilimumab in metastatic melanoma	Melanoma	Stereotactic body radiotherapy, ipilimumab	NCT02406183

30	Lenalidomide maintenance therapy for multiple myeloma	Multiple myeloma	Lenalidomide	NCT01675141

31	Ipilimumab and all-trans retinoic acid combination treatment of stage IV melanoma	Melanoma	All-trans retinoic acid ipilimumab	NCT02403778

32	Study evaluating the influence of LV5FU2 bevacizumab plus ANAKINRA Association on Metastatic Colorectal Cancer	Metastatic colorectal cancer	ANAKINRA	NCT02090101

33	A phase I/Ib study of AZD9150 (ISIS-STAT3Rx) in patients with advanced/metastatic hepatocellular carcinoma	Advanced adult hepatocellular carcinoma	AZD9150	NCT01839604
		Hepatocellular carcinoma metastatic		

34	AZD9150 with MEDI4736 in patients with advanced pancreatic, non-small lung and colorectal cancer	Malignant neoplasm of digestive organs intestinal tract; primary malignant neoplasm of respiratory and intrathoracic organ carcinoma	MEDI4736	NCT02983578
			AZD9150	

35	Study to assess MEDI4736 with either AZD9150 or AZD5069 in advanced solid tumors and relapsed metastatic squamous cell carcinoma of head and neck	Advanced solid tumors and metastatic squamous cell carcinoma of the head and neck	MEDI4736	NCT02499328
			AZD9150	
			AZD5069	
			Tremelimumab	

## Inhibition of MDSC-Mediated Immunosuppression

In preclinical mouse models, it has been demonstrated that inhibitors of phosphodiesterase-5, sildenafil, and tadalafil significantly inhibited the MDSC functions by the downregulation of iNOS and ARG1 activities, leading to the activation of antitumor immunity and the prolongation of survival of tumor-bearing mice (69–71). Recent clinical trials with tadalafil in patients with head and neck squamous cell carcinoma and melanoma confirmed this positive effect (72–74). It was shown that decreased amounts of MDSC and their immunosuppressive pattern correlated with an increased T cell reactivity and improved clinical outcome of advanced cancer patients.

A class I histone deacetylase inhibitor, entinostat, has been recently evaluated in several preclinical tumor models for its ability to affect MDSC functions (75, 76). The authors demonstrated that entinostat reduced the expression of ARG1, iNOS, and COX2 in both M- and PMN-MDSC subsets. In addition, they observed a strong reduction of tumor-infiltrating macrophages, suggesting a strong effect of this drug on the innate immunity. Interestingly, the combination of entinostat with anti-PD-1 antibodies significantly increased survival and delayed tumor growth in mice with LLC and renal cell carcinoma as compared to the treatment with anti-PD-1 antibodies alone. A combined therapy with nivolumab and entinostat in renal cell carcinoma patients is now planned.

A further promising way to target MDSC is the blockade of the activation of STAT3, which is a main transcription factor for immunosuppressive activity in myeloid cells (77). In the past, a number of clinical trials have been performed to target STAT3 with small molecular inhibitors with a limited efficacy and broad side effects (78). Recently, a new possibility to target STAT3 has been tested, in which STAT3 siRNA or decoy oligonucleotides were used to interfere with STAT3 mRNA (78). At the moment, several STAT3 oligonucleotide inhibitors, in particular AZD9150, were applied in the combination with immune checkpoint inhibitors in the frame of the phase I/II clinical trial (Table 1). In another approach, STAT3 siRNA or decoy oligonucleotides were coupled to CpG oligonucleotides, which are well-known agonists of TLR9 (79, 80). By this technique, a selective delivery of the drugs to TLR9-positive cells was ensured. Upon the treatment, TLR9-expressing myeloid cells (in particular, PMN-MDSC) displayed a decreased immunosuppressive activity, whereas TLR9-positive tumor cells lost the resistance to apoptosis *via* the STAT3 signaling (79, 80).

A further possibility to target MDSC is the modulation their metabolic pathways (81, 82). It was shown that tumor-infiltrating MDSC displayed an upregulation of the fatty acid translocase, CD36, which resulted in an increased uptake and oxidation of fatty acids. Accumulated lipids were reported to further increase an immunosuppressive capacity of MDSC in a STAT3- and STAT5-dependent manner (83). Pharmacological inhibition of the fatty acid oxidation decreased the immunosuppressive capacity of MDSC and in combination with low-dose chemotherapy and adoptive cellular therapy resulted in antitumor effect in LLC and colon adenocarcinoma mouse models (81).

## Blocking MDSC Trafficking

Myeloid-derived suppressor cells exhibit their main immunosuppressive activity within the TME. Therefore, intensive investigations were performed to block the migration of MDSC to the tumor site. Chemokine receptors are a key driving force for the migration of immune cells (84). Myeloid cells (in particular, MDSC) express C-X-C motif chemokine receptor (CXCR) 2 (85). The main ligands for CXCR2 are C-C motif chemokine ligand (CCL)2 and CCL5, which are elevated in the TME (86, 87). To block the CXCR2-CCL2 interaction, tumor-bearing mice were treated with the chemotherapeutic drug docetaxel combination with a CXCR2 antagonist, showing a significant therapeutic effect (88).

Another chemokine receptor CCR5, which is expressed on a broad spectrum of immune cells (84), interacts with its ligands CCL3, CCL4, and CCL5 (89). Interestingly, the patients with a mutated CCR5 variant were reported to be resistant to the prostate cancer development (90). Furthermore, CCR5 has a critical role in tumor progression since it has been shown that the CCR5–CCL5 axis supported tumor growth, invasion, and migration of MDSC to the tumor site (87, 91). By targeting the CCR5-CCR5 ligand interaction, tumor growth and invasiveness could be suppressed in pancreatic, colorectal, prostate, and breast cancer (92–94).

In a spontaneous *Ret* transgenic mouse melanoma model, we have demonstrated that the tumor progression correlated with the accumulation of CCR5^+^ MDSC in the TME that displayed significantly stronger immunosuppressive capacity than their CCR5^−^ counterpart (87). By blocking the CCR5–CCR5 ligand interaction with a mCCR5-Ig fusion protein, the survival of melanoma bearing mice was significantly improved as compared to the control group. Importantly, it was also shown that the frequency of CCR5^+^ M-MDSC and CCR5^+^ PMN-MDSC was increased in the peripheral blood of melanoma patients and that CCR5^+^ M-MDSC accumulated in melanoma lesions (87). Similar to the situation in melanoma bearing mice, CCR5^+^ MDSC from melanoma patients displayed an increased immunosuppressive pattern compared to the CCR5^−^ MDSC subset. Taken together, targeting CCR5 on MDSC could be applied not only to prevent the MDSC migration and accumulation in the TME but also to reduce MDSC immunosuppressive functions in cancer patients (87, 91).

## Depletion of MDSC

The number of MDSC in tumor-bearing hosts could be reduced by (i) the normalization of myelopoiesis, (ii) the inhibition of the conversion of IMC into MDSC, and (iii) the differentiation of MDSC into mature myeloid cells like DC or macrophages. All-trans retinoic acid (ATRA) seems to be a very promising agent for these approaches. ATRA is a vitamin A derivative binding to the retinoic acid receptor. By blocking the retinoic acid signal transduction, MDSC could differentiate into DC and macrophages (95). In addition, it was described that administration of ATRA led to the downregulation of ROS production in MDSC by activating the extracellular-signal regulated kinase (ERK)1/2 pathway (96). In a completed clinical trial, ATRA was applied in metastatic renal carcinoma patients in combination with the IL-2 administration (97). The frequency of MDSC was significantly decreased, and the ratio between DC and MDSC was much higher than in the untreated group. In a second clinical trial with late stage small cell lung cancer patients, ATRA was used together with a DC vaccine against p53 (98). The outcome confirmed the inhibitory effect of ATRA on the frequency of circulating MDSC. The combination of the DC vaccine and ATRA resulted in the development of p53-specific CD8^+^ T cells. It should be mentioned that ATRA was used in many other clinical trials with inhibitory effects on tumor progression; however, MDSC were not evaluated in these trials, and the positive effect was linked to other mechanisms.

Since tumor-derived EV were reported to induce the conversion of non-immunosuppressive IMC into MDSC and further activated their immunosuppressive functions (26, 27), the inhibitors of the EV release from tumor cells were tested in mice-bearing CT26 colon carcinoma (30). It was demonstrated that the treatment of these mice with dimethyl amiloride or omeprazole reduced EV content in serum that was associated with the reduction of MDSC expansion and immunosuppressive activity (30).

Clinical trials with tyrosine kinase inhibitors (such as sunitinib) revealed that these agents could target MDSC. Since sunitinib could block VEGF and c-kit signaling, which are involved in the generation of MDSC (99), its effect on MDSC from cancer patients was evaluated. Sunitinib treatment of metastatic renal cell carcinoma patients was reported to decrease the number of circulating MDSC (100, 101). Interestingly, M-MDSC from treated patients displayed a reduced STAT3 activation and ARG1 expression that was accompanied with an elevated activity and proliferation of CD8 T cells. However, no significant prolongation of the overall survival was observed.

Other chemotherapeutics such as gemcitabine and 5-fluorouracil were shown to induce selectively apoptosis of MDSC in the spleen and TME in several mouse tumor models (102–104). Interestingly, both chemotherapeutic agents displayed no significant effect on the frequencies of T cells, NK cells, DC, and B cells. It was also shown that gemcitabine reduced the frequency MDSC and Treg as well TGF-β1 level in the peripheral blood of pancreatic cancer patients (103). Similar to the preclinical observation, gemcitabine has no effect in effector T cells. In a clinical trial, gemcitabine treatment of pancreatic cancer patients resulted in a dramatic decrease in PMN-MDSC (103). An application of 5-fluorouracil in the preclinical mouse model and colorectal cancer patients affected MDSC, leading to the immune recovery and tumor regression (104). Administration of another chemotherapeutic, docetaxel, induced a decrease of tumor burden in a preclinical mouse model of mammary carcinoma (105). This beneficial effect was accompanied by the conversion of MDSC into a M1-like cells characterized by the upregulation of CCR7 (105). The effect of doxorubicin on MDSC in mammary cancer models was also investigated (106). The treatment of these mice with doxorubicin led to the reduction of MDSC frequencies in the spleen, peripheral blood, and tumors. Furthermore, the immunosuppressive activity of residual MDSC was impaired. The depletion of MDSC resulted in the enhancement of granzyme B and IFN-γ production by effector T and NK cells (106). Moreover, this study demonstrated that MDSC isolated from patients were also sensitive to doxorubicin treatment *in vitro* (106).

Using *Ret* transgenic melanoma mouse model, we demonstrated that the administration of ultra-low, non-cytotoxic doses of paclitaxel induced the reduction of MDSC numbers and immunosuppressive functions (107). This effect was associated with an inhibition of the p38 MAPK pathway as well as the production of TNF-α and S100A9 in MDSC. Treated mice showed elevated activity of CD8 T cells, which correlated with the prolongation of mouse survival (107). In addition, it was reported that the treatment of MDSC *in vitro* with ultra-low concentrations of paclitaxel stimulated their differentiation into DC (108).

## Future Perspectives

Tumor cells developed multiple mechanisms to evade the immune system and to progress. One of the key mechanisms is the establishment of an immunosuppressive TME, where MDSC play a crucial role. By altering MDSC function and biology, various preclinical and clinical studies showed a beneficial effect. This suggests that MDSC targeting could be a promising strategy to apply together with existing immunotherapeutic strategies such as boosting the immune system by vaccination or negative immune checkpoint inhibitors. Thus, combining gemcitabine with a DNA vaccination induced a strong antitumor immune response accompanied by a reduced self-tolerance in a preclinical HER2-expressing mouse tumor model (109). Furthermore, another preclinical study showed that the administration of sunitinib with an HPV vaccination resulted in a tumor-free survival in 75% mice in the HPV-expressing tumor model (110). In addition, a clinical trial was initiated in stage IV melanoma patients, by whom ATRA was applied together with ipilimumab (111). This trial and many other starting combinatorial approaches will help to develop an efficient strategy for the treatment of cancer patients.

## Author Contributions

VF: writing, review, and revision of the manuscript and revision of the figures. XH: preparing the figures. RW, PA, and JU: review and revision of the manuscript. VN: preparing the table. CG: revision of the manuscript. VU: writing, review, and revision of the manuscript and revision of the table and figures.

## Conflict of Interest Statement

The authors declare that the research was conducted in the absence of any commercial or financial relationships that could be construed as a potential conflict of interest.

## Abbreviations

ARG1, arginase-1; ATRA, all-trans retinoic acid; bFGF, basic fibroblast growth factor; CD, cluster of differentiation; CCL, C-C motif chemokine ligand; CCR, C-C motif receptor; COX, cyclooxygenase; CXCL, C-X-C motif ligand; CXCR, C-X-C motif receptor; DC, dendritic cell; ERK, extracellular-signal regulated kinase; EV, extracellular vesicles; HSP, heat shock protein; IDO, indoleamine 2,3-dioxygenase; IFN, interferon; IL, interleukin; iNOS, inducible nitric oxide synthase; LLC, Lewis lung carcinoma; LOX, lectin-type oxidized low-density lipoprotein receptor; IMC, immature myeloid cells; M, monocytic; MCP, monocyte chemoattractant protein; MDSC, myeloid-derived suppressor cells; MMP, matrix metalloproteinases; NO, nitric oxide; NSCLC, non-small cell lung cancer; PD-1, programmed death receptor; PD-L1, programmed death ligand 1; PMN, polymorphonuclear; ROS, reactive oxygen species; STAT, signal transducer and activator of transcription; TCR, T cell receptor; TGF, transforming growth factor; TLR, toll-like receptor; TME, tumor microenvironment; TNF, tumor necrosis factor; Treg, regulatory T cells; VEGF, vascular endothelial growth factor.
